# Natural variation in female reproductive hormones does not affect contrast sensitivity

**DOI:** 10.1098/rsos.171566

**Published:** 2018-02-28

**Authors:** Abigail L. M. Webb, Paul B. Hibbard, Rick O'Gorman

**Affiliations:** Department of Psychology, University of Essex, Wivenhoe Park, Essex CO4 3SQ, UK

**Keywords:** menstrual cycle, contrast sensitivity, cycle shift hypothesis

## Abstract

Evidence suggests that females experience adaptive shifts in facial preferences across the menstrual cycle. However, recent discussions and meta-analyses suggest that these findings are equivocal. A previously unexplored question is the extent to which shifts in female preferences are modulated by hormone-dependent changes occurring in low-level vision, such as visual sensitivity. This mechanistic approach has been a novel method for investigating the extent to which complex perceptual phenomena are driven by low-level versus higher-level perceptual processes. We investigated whether the contrast sensitivity function—an early dimension of vision—is also influenced by variation in female reproductive hormones. Visual contrast thresholds were measured for 1, 4 and 16 cycles/degree gratings during the ovulatory, luteal and menstrual phases of the menstrual cycle in naturally cycling women, and women using oral contraceptives. Male participants were tested at similar time intervals. Results showed that visual contrast sensitivity does not differ according to sex, or use of oral contraception, nor does it vary relative to hormonal shifts across the menstrual cycle. These findings suggest that shifts in female preferences are not driven by changes in visual sensitivity, and are therefore likely attributable to changes in higher-level perception or cognition.

## 1. Introduction

The cycle shift hypothesis [1] suggests that certain aspects of females' sexual preferences, including judgements of facial attractiveness, are not fixed, but vary with fluctuations in oestrogen that occur across the menstrual cycle. Oestrogen and conception probability climb from day 8 of the cycle towards peak fertility on day 14, declining markedly afterward [2,3]. This fertile period is associated with a shift of females’ preferences towards prospective mates with presumed physical indicators of ‘good genes'; traits that are adaptive for offspring to inherit. During the fertile period, women display greater interest in symmetrical male faces—a physical feature that is thought to be a cue to developmental health [4]—particularly when rating these faces for their attractiveness as short-term sexual partners [5]. Fertile women's preferences also extend to physical cues that signify immunocompetence [6,7], including preferences for faces of males with higher levels of testosterone [8], darker facial skin tone [9] and synthetically exaggerated facial masculinity [10].

These preferences are more evident in the period of highest fertility, during the days preceding ovulation [11,12]. In the days following ovulation, progesterone levels rise and preferences reorient towards cues suggestive of males' capacity for parental investment [13]. Fertility-dependent shifts in preferences for distinct facial cues of health and sex-typicality are thought to maximize females’ chances of producing healthy and reproductively successful offspring. This notion is rooted in the idea that females face particular evolutionary pressures that make choosing the optimal partner to provide the other half of her offspring's genes a formidable task (see review by Kokko *et al*. [14]). Cyclic shifts in visual preferences are therefore thought to guide females' mating efforts towards partners who are most likely to instil their offspring with good genes, or provide qualities of parental investment [15].

A notable limitation of the cycle shift hypothesis is the variability in findings for a relationship between female fertility and perceived attractiveness when making mate preferences. This has been discussed at length across several meta-analyses, creating two polarized bodies of research that fervently support or reject the validity of the cycle shift hypothesis [16–20]. This controversy has tended to focus on the way in which the timing of the menstrual cycle is established and the role of moderating factors, such as whether attractiveness is judged in the context of a short-term or long-term relationship.

There has also been an emphasis on the effects on judgements of attractiveness, which depend on complex, semantically meaningful visual information. While there has been relatively little research into menstrual cycle effects on low-level visual processing [21], there is some evidence for changes in sensitivity across the cycle. Wong & Tong [22] used a two-flash threshold, which is a measure of the minimum temporal separation at which two flashed stimuli can be distinguished from a single target. In naturally cycling women, they found lower thresholds around, and just before, ovulation, which they interpreted as a result of increased arousal and cortical activity at this time. No menstrual cycle effect was found for women using hormonal contraceptives [22]. Maguire & Byth [23] measured the McCullough effect, an orientation-contingent colour aftereffect, and found a greater effect pre-menstrually, again only for naturally cycling women. This was taken as evidence that the size of the effect is inversely related to cholinergic activity [23]. Parlee [24] reviewed a range of studies of visual sensitivity across the menstrual cycle [24] (DeMarchi & Tong [25], Diamond *et al.* [26], Ward *et al*. [27], Barris *et al*. [28], Scher *et al*. [29]). The consensus of these studies is that simple visual sensitivity tends to increase around ovulation. Epting & Overaman [30] assessed the effect of the menstrual cycle on a range of cognitive and motor skills tasks. Some of these tasks (mental rotation, rod and frame and water level tasks) tend to be performed better by men, while others (finger tap and spatial array tasks, and the Purdue pegboard) tend to be performed better by women. They found no effects of menstrual cycle on performance of any of these tasks [30]. Johnson & Petersik [31] measured contrast sensitivity for static and drifting (5 Hz) gratings at three spatial frequencies (2, 4 and 16 cycles/degree (cpd)). They found that, for normally cycling women, contrast sensitivity tended to increase at the postovulatory phase of the cycle, and that this effect was strongest for static 4 cpd stimuli [31].

These results are generally consistent with Kopell's [32] proposal that ovulation may be accompanied by a global peak in visual arousal. However, the findings from these studies are equivocal due to methodological inconsistencies. These include test sessions that occur during a single phase of the menstrual cycle, measurements that take place during days that do not correspond to the period of highest fertility, and non-stringent inclusion of control groups, such that there is little consistency in the kinds of hormonal contraceptives used, or a lack of a non-ovulating control group altogether. This may be because such psychophysical studies are seldom concerned with the evolutionary relevance of the menstrual cycle effects, and so are therefore less likely to adopt methodological techniques used in studies from evolutionary psychology. If it is the case that visual perception in women is affected by fertility status, then menstrual cycle phase may be an important factor to consider during interpretation of results. To date, Johnson & Petersik [31] provide the most detailed measure of contrast sensitivity, where visual contrast thresholds were measured across consecutive days during a single cycle. However, this was a preliminary study of four participants, and did not include a reliable comparison of women using oral contraceptives that are known to inhibit naturally occurring ovulation. Additionally, average contrast thresholds were calculated for the days of the cycle on which testing did not take place. As female reproductive hormones change on a daily basis across the cycle, replacing actual measurements with calculated averages may mean that hormone-related changes in contrast sensitivity were not accurately recorded.

Given the controversy of results on the cycle shift hypothesis, and the focus on complex, socially significant judgements, it is important to also consider the extent to which low-level visual sensitivity might also vary across the menstrual cycle. Not only is this an important question in its own right, but such changes in sensitivity might also contribute to variation in higher-level judgements, thus providing a potential mechanism for these effects. One possibility is that the low-level processing of visual attributes is unchanged, and that the cycle shift represents a change in the way in which higher-level factors, such as averageness, symmetry and sexual dimorphism, are weighed in establishing attractiveness. As an alternative, variations in low-level visual processing across the cycle could influence attractiveness judgements by changing the information available to these higher-level mechanisms. Incorporating the influence of such low-level visual mechanisms on apparently high-level perceptual phenomena has recently enhanced understanding of the visual mechanisms underlying the threat bias in the perception of facial expressions [33].

The present experiment explored the possibility that contrast sensitivity might vary across the menstrual cycle, and that this might contribute to cycle shifts in visual mate preferences. It is possible that changes in visual sensitivity at this level could directly affect the way in which attractiveness judgements are made. The contrast sensitivity function describes the relative sensitivity of the visual system to information at different scales and orientations [34], and influences the relative salience of the information available for judgements of attractiveness. These judgements can be made successfully with relatively low spatial frequency information, below 7 cpd [35,36]. Judgements of facial symmetry differ from low-level symmetry judgements, in that they depend on the spatial scale of information, being particularly associated with medium and high spatial frequencies [37]. This means, for example, that a shift in the contrast sensitivity function could alter the salience of facial symmetry information. In turn, this could influence how attractiveness is judged. Research also suggests that contrast sensitivity may be influenced by activity in the GABAergic system; an effect that is strongly associated with menstrual cycle phase and use of hormonal contraception [38–40]. If it is the case that cyclic changes in the GABAergic system affect contrast sensitivity, then we may expect to observe changes in contrast sensitivity across the menstrual cycle. Together, these findings demonstrate the way in which aspects of higher-level perceptual behaviours, such as judgements of attractiveness, are influenced by low-level visual processes and basic image properties associated with distinct facial features. However, in contrast, studies of the cycle shift hypothesis have not established the proximal causes of the effect, and whether these occur at a low or high level of visual processing.

The purpose of this current study was therefore to provide an extensive measure of contrast sensitivity across the menstrual cycle in naturally ovulating women, users of combined oral contraceptives, and men. Separate participant groups were selected in order to investigate sex differences in contrast sensitivity between men and women, and to establish whether potential cycle effects are confined only to female participants experiencing naturally occurring ovulation. Of multiple candidate measures, visual contrast sensitivity was selected because it is well recognized as a dimension of early visual processing and a reliable measure of visual sensitivity that may directly influence the information available for judgements of attractiveness [35–37,41,42]. If fertile women experience enhanced interest in particular facial cues, it may be that this effect is modulated by a mid-cycle peak in contrast sensitivity.

## 2. Materials and methods

### 2.1. Participants

Forty-nine participants took part in the experiment. Twenty-one of these were naturally cycling women who were not using any hormonal oral contraceptives (mean age: 21.6 years, s.d. 3.8). Fourteen were women using combined oral contraceptives at the time of testing (mean age: 20 years, s.d. 1.3). For these participants, artificial levels of both oestrogen and progesterone inhibited naturally occurring ovulation. Brands of combined oral contraception included Yasmin, Rigevidon, Loette 28, Marvelon, Yaz and Microgynon. Fourteen participants were male (mean age: 21.5 years, s.d. 4.3). All participants were recruited on a voluntary basis, taking part in exchange for either monetary reward or to acquire credits for an undergraduate research module.

Female participants met a stringent set of eligibility criteria. Eligibility was established via an online pre-screening questionnaire to ensure participants experienced predictable and regular cycles constituting of no more than 35 days, and no breastfeeding or use of emergency contraception or hormonal contraceptives 3 months prior to testing. Participants using combined oral contraceptives were required to meet the same criteria regarding cycle length.

No participants reported using medications that were considered to influence visual sensitivity. All participants had normal or corrected-to-normal vision.

### 2.2. Stimuli and apparatus

Stimuli were sinusoidal Gabor patches with a spatial frequency of 1, 4, or 16 cpd. The standard deviation of the Gabors was 4.8 times the wavelength of the sinusoid. This meant that the size of the stimuli decreased with spatial frequency, but ensured that the number of grating cycles was constant. The orientation of the sinusoid on each trial was ±45° away from vertical, either clockwise or anticlockwise. Six Michelson contrast levels were used, each presented 40 times in a randomized order. Michelson contrast refers to the lightest (*L*_MAX_) and darkest (*L*_MIN_) points in an image, where these are standardized on a 0–100% scale

$${\text{C}}_{\mathrm{Michelson}}=100\cdot \left(\frac{{\text{L}}_{\mathrm{MAX}}-{\text{L}}_{\mathrm{MIN}}}{{\text{L}}_{\mathrm{MAX}}+{\text{L}}_{\mathrm{MIN}}}\right)\%.$$

**Figure 1.**
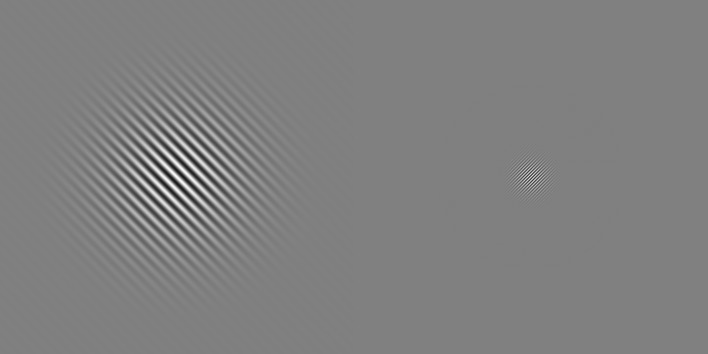
Examples of Gabor gratings representing low (1 cpd), mid-range spatial frequencies (4 cpd), at the two orientations used.

For 1 cpd gratings, the Michelson contrast levels used were 0.1, 0.2, 0.3, 0.4, 0.5 and 0.75%. For 4 cpd gratings, the contrast levels used were 0.1, 0.15, 0.2, 0.3, 0.4 and 0.5%. For 16 cpd gratings, the contrast levels used were 2.0, 3.0, 4.0, 5.0, 6.0 and 7.0%. These contrasts were selected after extensive pilot measures, run in order to establish the appropriate contrast variations for each spatial frequency. Examples of these stimuli are shown in figure 1. Stimuli were generated and presented using Matlab and the Psychophysics Tool box extensions [43–45].

Stimuli were presented using a VIEWPIXX 3D monitor (52 cm **× **29 cm), viewed from a distance of 65 cm. The screen resolution was 1920 × 1080 pixels, with a refresh rate of 120 Hz and an average luminance of 50 cd m**^−^**^2^. Each pixel subtended 1.43 arc min. Stimuli were presented at 10 bit resolution. Participants' responses were recorded using the RESPONSEPixx response box.

### 2.3. Procedure

Participants were tested individually in a quiet room and informed of the nature of the study. All participants completed three experimental test sessions across a single cycle, scheduled to correspond to three critical phases of their menstrual cycles. This included the early follicular phase (or menses) occurring between days 1 and 7; the ovulatory phase, occurring between days 11 and 14; and the luteal phase, occurring between days 17 and 28. Test sessions for male participants were spaced one week apart to reflect similar time intervals. Cycle position was estimated using the backwards-counting [17,46] method to calculate the ‘fertile window’ for each participant. This methodological procedure was adopted for its reported reliability for approximately calculating high fertility days of the cycle [17]. Using the date of females' previous and next expected menstrual onset, the ovulatory phase—also the first test session—was calculated to take place during days 11–14 of the cycle. Following this, female participants were recorded 7–10 days later during their estimated luteal phase, followed by the final phase that was calculated to take place during the menstrual phase of their menstrual cycle. During the final test session, female participants verbally confirmed menstrual onset. Given that the pre-screen questionnaire ensured that participating women experienced cycles of no more than 35 days, confirmation of menstrual onset suggested that calculations of the fertile period two to three weeks prior were accurate. Male participants were tested during similar time intervals.

During each of the 3 sessions, 3 blocks of 240 trials were presented to participants. For each trial, a fixation cross was presented for 250 ms, followed by the stimulus for 500 ms. Participants used the left or right button to indicate whether the Gabor stimulus was tilted leftwards or rightwards from vertical. After responding, a fixation cross appeared before the onset of the next trial. The trials for each spatial frequency were presented in three separate blocks; the order in which these were presented was randomized across participants. Within a block, each contrast level was presented 40 times, in a randomized order. Experiments were performed in a dimly lit laboratory.

During each testing session, stimuli of a single spatial frequency were presented at six contrast levels. Each contrast level was presented 40 times. We recorded the proportion of times that the participant correctly reported the orientation of the stimulus for each level. This was used to create a psychometric function. A cumulative Gaussian function was fit to these data and was used to determine a contrast detection threshold. This was defined as the contrast required for the participant to correctly identify the orientation of the stimulus on 75% of trials.

## 3. Results

**Figure 2.**
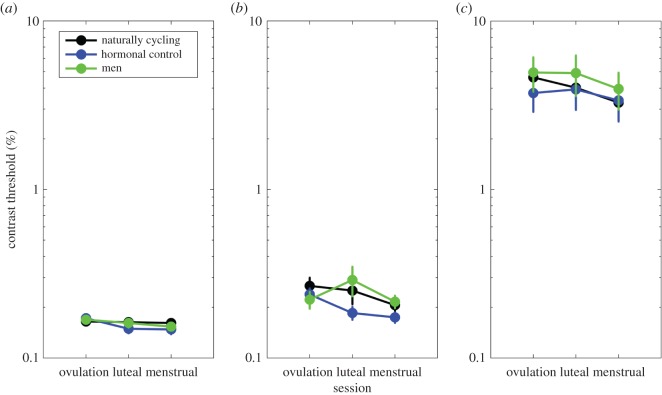
(*a*) Contrast thresholds for low (1 cpd) gratings, at each of the three test sessions. No significant group differences were found. (*b*) Data for mid-range (4 cpd) gratings. No significant group differences were observed, showing that contrast sensitivity does not vary according to between participant sex, or between naturally cycling women and users of oral contraceptives. (*c*) Data for high (16 cpd) stimuli, where again no significant differences between participant groups were observed.

A 3 (participant group) × 3 (frequency) × 3 (test session) mixed ANOVA was used to analyse the data. Findings showed that visual contrast thresholds varied with spatial frequency (*F*_2, 92_ = 58.28, *p* < 0.001, $\eta_{p}^{2}=0.55$), where visual thresholds were lower for low-frequency gratings. *T*-tests revealed that contrast thresholds were lower for 1 cpd (mean threshold 16%, s.d. < 0.001) and 4 cpd gratings (mean threshold 23%, s.d. 0.001, *t*_146_ = −6.38, *p* < 0.001) than for 16 cpd gratings. This is shown in figure 2*a*–*c*. These findings show that contrast sensitivity decreases with increasing spatial frequency. This is consistent with the previous literature [21], where under normal photopic conditions less contrast is required to identify lower frequency stimuli within the range used here. It is also important to note, because contrast sensitivity is known to vary as a function of observer age [47] that participant age data were not subjected to statistical analyses on the basis that the age range of participants (female age range: 18–26, male age range: 18–35) did not fall within an age group where contrast sensitivity is expected to decline.

In terms of gender differences and cycle-shift effects, no significant effect of participant group was found (*F*_2, 46_ = 0.261, *p* = 0.771, $\eta_{p}^{2}=0.01$). This shows that visual contrast thresholds do not differ between men and women, and that naturally occurring ovulation is not associated with visual sensitivity that is higher than that of users of combined oral contraception, whose visual sensitivity is thought to be inhibited by synthetic reproductive hormones [28].

A significant effect of test session (*F*_2, 92_ = 4.94, *p* = 0.009, $\eta_{p}^{2}=0.09$) showed differences in contrast thresholds across the three experimental sessions. Figure 2 shows this effect of improvement by a gradual decrease in visual contrast thresholds across experimental sessions.

To ensure that hormone-related differences in contrast thresholds across the three test sessions were not masked by practice effects, three additional two-way between-participants ANOVAs were performed for each test session. The first two-way ANOVA took data from the ovulatory phase, at session 1. The effect of frequency was maintained (*F*_2, 4_ = 55.61, *p* < 0.001, $\eta_{p}^{2}=0.54$). No effect of group (*F*_2, 46_ = 0.34, *p* = 0.71, $\eta_{p}^{2}=0.01$) or group by frequency interaction (*F*_4, 92_ = 0.37, *p* = 0.746, $\eta_{p}^{2}=0.01$) was observed. The second two-way ANOVA took data from the luteal phase, at session 2. The effect of frequency was maintained (*F*_2, 4_ = 47.9, *p* < 0.001, $\eta_{p}^{2}=0.51$), and no significant effect of group (*F*_2, 46_ = 0.29, *p* = 0.746, $\eta_{p}^{2}=0.01$) or group by frequency interaction (*F*_4, 92_ = 0.24, *p* = 0.91, $\eta_{p}^{2}=0.01$) was found. Finally, the third two-way ANOVA took data from the menstrual phase, at session 3. The effect of frequency was maintained (*F*_2, 4_ = 52.54, *p* < 0.001, $\eta_{p}^{2}=0.53$), and no significant effect of group (*F*_2, 46_ = 0.20, *p* = 0.818, $\eta_{p}^{2}=0.00$) or group by frequency interaction (*F*_4, 92_ = 0.20, *p* = 0.939, $\eta_{p}^{2}=0.00$) was found.

Together, these results demonstrate internal reliability in showing that they accurately measure the contrast sensitivity function [34]. However, no sex differences in visual contrast thresholds were identified for male and female participants. Additionally, no effect of changing oestrogen levels was identified; naturally cycling women do not experience a mid-cycle peak in contrast sensitivity, nor is this effect inhibited in those using combined oral contraceptives. Based on the measured standard deviations, improvements in contrast sensitivity of around 10% (1 cpd), 25% (4 cpd) or 55% (16 cpd) would have been detectable with 80% power for the normally cycling group. These compare with the practice effects observed for this group of 2% (1 cpd), 23% (4 cpd) and 29% (16 cpd).

## 4. Discussion

The purpose of the present experiment was to investigate the extent to which cycle shifts in females’ facial preferences might be underpinned by low-level factors of early visual processing. We considered whether the enhanced preference for certain facial characteristics reported during mid-cycle occurs as a result of hormone-related changes in visual sensitivity across the menstrual cycle. Contrast sensitivity was measured across the menstrual cycles of naturally ovulating women and women whose ovulation had been artificially inhibited by hormonal contraceptives. Test sessions took place over a single cycle, on three separate occasions corresponding to the ovulatory, luteal and menstrual phases of the menstrual cycle, and similar time intervals for male participants. Results were consistent with existing psychophysical literature, showing the typical contrast sensitivity function and a degree of perceptual learning had been accurately recorded [34,48]. Previous studies have suggested that there might be sex differences in contrast sensitivity [49,50]. However, the present study provided an extensive measure of contrast sensitivity between men and women, and found no evidence for such an effect; visual contrast thresholds do not differ according to participants' sex. Importantly, no variability in contrast thresholds was identified across the menstrual cycles of naturally ovulating women compared to those whose ovulation had been inhibited through the use of hormonal contraceptives.

Many recent discussions of the cycle shift hypothesis have focused primarily on the variability and reliability of findings. These discussions have taken place across several meta-analyses that extensively argue opposing positions on the matter. However, missing from the debate is the way in which cycle effects might be understood in terms of their underlying mechanisms. The importance of understanding a phenomenon's mechanisms of control is a well-recognized tenet of evolutionary psychology [51]. This method has, for example, recently been used to provide a tractable understanding of the way in which high-level perceptual processing, such as the threat bias in face perception, is driven by low-level processes occurring in early vision [33]. Results from the present study provide an extensive measure of the relationship between female reproductive hormones and contrast sensitivity, and suggest that cycle changes in female preferences are unlikely to occur as a result of hormonally driven changes operating in this aspect of early visual processing. These findings have novel relevance in progressing the current understanding of the relationship between female visual perception and reproductive hormones. The finding that female fertility is not associated with visual sensitivity suggests that variation in female preferences likely occurs not because of changes in contrast sensitivity. It remains possible that other aspects of low-level vision, for example sensitivity to symmetry, might be modulated by variations in reproductive hormones, and therefore contribute to changes in visual preferences. Alternatively, changes in preferences might relate to higher-level perception. This latter possibility is consistent with the arguments from previous studies, suggesting that an element of context-dependent factors, such as relationship status, plays an important role during mate selection [13]. This indicates that the mechanisms of control may be operating in areas involved in cognitive appraisal and decision-making. Until recently, this has been an understudied topic within discussions from both the psychophysical and evolutionary psychology literatures. Overall, these results suggest that mechanisms underlying cycle shifts in female facial preferences do not operate in low-level vision, rather, that they are likely to occur as a result of shifts that occur in higher-level visual perception.

## 5. Conclusion

We found no evidence for changes in visual sensitivity associated with menstrual cycle variations in female fertility. These results suggest that variation in female preferences when judging the attractiveness of faces likely occurs not because of low-level factors, but because of changes that occur in higher-level perception. This is consistent with the arguments from previous studies, suggesting that an element of context-dependent factors, such as relationship status, plays an important role during mate selection [13]. This indicates that the mechanisms of control may be operating in areas involved in cognitive appraisal and decision-making. Until recently, this has been an understudied topic within discussions from both the psychophysical and evolutionary psychology literatures. Psychophysical studies have previously shown that aspects of vision may be influenced by hormonal changes in women [22–29], but because these studies were not concerned with the evolutionary relevance of such changes, such as fertility effects, full cycles and participant groups were not always part of their experimental designs. Equally, evolutionary studies that do include such specific experimental designs focus on higher-level aspects of perception, such that input from low-level vision is not the focus of investigation [14–20]. With these limitations combined, it has remained unclear the extent to which female reproductive hormones influence early visual processing, and how these effects may contribute to understanding shifts in preferences reported in the evolutionary psychology literature. The present results address these issues, and suggest that mechanisms underlying cycle shifts in female facial preferences do not operate in low-level vision, rather, that they are likely to occur as a result of shifts that occur in higher-level visual perception.

## Data accessibility

Data are available from the Dryad Digital Repository (https://doi.org/10.5061/dryad.rv6b7) [52]. Contrast sensitivity data: publicly available at Figshare (https://doi.org/10.6084/m9.figshare.4791943.v1).
